# Analysis of the mitochondrial maxicircle of *Trypanosoma lewisi*, a neglected human pathogen

**DOI:** 10.1186/s13071-015-1281-8

**Published:** 2015-12-30

**Authors:** Ruo-Hong Lin, De-Hua Lai, Ling-Ling Zheng, Jie Wu, Julius Lukeš, Geoff Hide, Zhao-Rong Lun

**Affiliations:** Center for Parasitic Organisms, State Key Laboratory of Biocontrol, School of Life Sciences and Key Laboratory of Tropical Diseases and Control of the Ministry of Education, Zhongshan School of Medicine, Sun Yat-Sen University, Guangzhou, The People’s Republic of China; Key Laboratory of Gene Engineering of the Ministry of Education, State Key Laboratory of Biocontrol, School of Life Sciences, Sun Yat-Sen University, Guangzhou, 510275 The People’s Republic of China; Institute of Parasitology, Biology Centre, Czech Academy of Sciences and Faculty of Science, University of South Bohemia, České Budějovice (Budweis), Czech Republic; Canadian Institute for Advanced Research, Toronto, Canada; Ecosystems and Environment Research Centre and Biomedical Research Centre, School of Environment and Life Sciences, University of Salford, Salford, UK

**Keywords:** *Trypanosoma lewisi*, Kinetoplast maxicircle, Mitochondrial DNA, RNA editing, Palindrome

## Abstract

**Background:**

The haemoflagellate *Trypanosoma lewisi* is a kinetoplastid parasite which, as it has been recently reported to cause human disease, deserves increased attention. Characteristic features of all kinetoplastid flagellates are a uniquely structured mitochondrial DNA or kinetoplast, comprised of a network of catenated DNA circles, and RNA editing of mitochondrial transcripts. The aim of this study was to describe the kinetoplast DNA of *T. lewisi*.

**Methods/Results:**

In this study, purified kinetoplast DNA from *T. lewisi* was sequenced using high-throughput sequencing in combination with sequencing of PCR amplicons. This allowed the assembly of the *T. lewisi* kinetoplast maxicircle DNA, which is a homologue of the mitochondrial genome in other eukaryotes. The assembly of 23,745 bp comprises the non-coding and coding regions. Comparative analysis of the maxicircle sequence of *T. lewisi* with *Trypanosoma cruzi, Trypanosoma rangeli*, *Trypanosoma brucei* and *Leishmania tarentolae* revealed that it shares 78 %, 77 %, 74 % and 66 % sequence identity with these parasites, respectively. The high GC content in at least 9 maxicircle genes of *T. lewisi* (*ATPase6*; NADH dehydrogenase subunits *ND3*, *ND7*, *ND8* and ND*9*; G-rich regions *GR3* and *GR4*; cytochrome oxidase subunit *COIII* and ribosomal protein *RPS12*) implies that their products may be extensively edited. A detailed analysis of the non-coding region revealed that it contains numerous repeat motifs and palindromes.

**Conclusions:**

We have sequenced and comprehensively annotated the kinetoplast maxicircle of *T. lewisi*. Our analysis reveals that *T. lewisi* is closely related to *T. cruzi* and *T. brucei*, and may share similar RNA editing patterns with them rather than with *L. tarentolae*. These findings provide novel insight into the biological features of this emerging human pathogen.

**Electronic supplementary material:**

The online version of this article (doi:10.1186/s13071-015-1281-8) contains supplementary material, which is available to authorized users.

## Background

The genus *Trypanosoma* belongs to the Kinetoplastea, which lies within the eukaryotic supergroup Excavata and comprises an assembly of mostly parasitic flagellated protists [1]. The best known trypanosomes are the human pathogenic *Trypanosoma brucei gambiense* and *T. b. rhodesiense* causing sleeping sickness in Africa, and *T. cruzi*, the causative agents of Chagas disease in South America. Other members of this genus are economically important animal parasites. *Trypanosoma lewisi* has long been recognized as a globally distributed obligatory parasite of rodents of the genus *Rattus*, transmitted by rat fleas and non-pathogenic to its natural hosts and humans [2]. This view has changed recently when human infections were reported [3] which culminated with a case of a fatal infection in an infant with a *T. lewisi*-like flagellate [4]. More importantly, the resistance of this parasite to the lysis by normal human serum was recently demonstrated [5]. These reports substantially raise the importance of this flea-transmitted trypanosome, which can now be considered a neglected human parasite [3, 6–8]. This is particularly important in developing countries where infants may have encountered direct contact with *T. lewisi*-infected rat fleas. More research on *T. lewisi* is warranted to establish a greater knowledge of the basic biology of this organism and its potential for contribution to human disease.

As a trypanosomatid flagellate, *T. lewisi* has an extensive mitochondrial DNA network composed of mutually interlocking DNA circles which are packed into a disk-shaped structure termed the kinetoplast DNA (kDNA). The kDNA consists of dozens of catenated maxicircles with species-specific sizes, ranging from 20 to 40 kb, and thousands of minicircles ranging from 0.5 to 10 kb, again in a species-specific manner [9–11]. The kDNA maxicircle is composed of two regions: a coding region carrying homologs of mitochondrial genes typical of other eukaryotes [12, 13] and a variable non-coding region, also known as the divergent region (DR), which may play a role in maxicircle replication [14]. The coding region contains two ribosomal RNA genes, fourteen protein-coding genes (*ND8*, *ND9*, *ND7*, *COIII*, *Cyb*, *ATPase6*, *MURF1* (now known to be *ND2* [15]), *ND1*, *COII*, *COI*, *ND4*, *ND3*, *RSP12*, *ND5*), four genes (*MURF2*, *MURF5*, *GR3* and *GR4*) of unknown function [16], and a few gRNAs (guide RNA) [16, 17]. The minicircles encode heterogenous guide RNA genes, which provide information for extensive RNA editing of maxicircle transcripts [18, 19]. This type of post-transcriptional modification entails the insertions, and less frequently deletions of uridines, using complex protein machinery and interactions [20–23].

Kinetoplast DNA has been established as a good taxonomic marker since it has a relatively fast rate of evolution [24]. Moreover, it is key to our understanding of RNA editing patterns and overall mitochondrial function. Hence, alterations in the kDNA may have a substantial impact on parasite development and the course of infection. It has been reported that *T. cruzi* isolates carrying a major deletion in the maxicircle-encoded *ND7* gene seem to cause only asymptomatic Chagas disease [25]. Furthermore, depletions and losses of the kDNA have been shown to play an important role in the evolution of trypanosomes, leading to the emergence of new species that are able to occupy new niches [26–28]. Therefore, the kDNA has a significant impact on the transmission, pathogenicity, development and evolution of trypanosomes.

Despite its potential importance as a zoonotic pathogen, no information is available on the maxicircle kDNA of *T. lewisi* [5]. Here, we present a well-annotated maxicircle sequence of the Chinese strain of *T. lewisi.* Comparative analyses with the maxicircle sequences of *T. brucei*, *T. cruzi*, *T. rangeli* and *Leishmania tarentolae* revealed high overall conservation of gene content and synteny. Our work provides a framework for future studies of the kDNA biology, RNA editing and evolution of *T. lewisi*. In addition, genetic information on the *T. lewisi* maxicircle can be used to design diagnostic molecular markers needed for the detection of this parasite in natural hosts as well as in humans.

## Methods

### Parasites, ultrastructure, isolation of kDNA and restriction endonuclease digestion

*Trypanosoma lewisi* CPO02 strain, isolated from a rat (*Rattus norvegicus*) trapped in Guangzhou [7], was used in this study. Trypanosomes were grown in Sprague Dawley rats and were harvested from blood by differential centrifugation. Briefly, red blood cells were pelleted at 180 x g for 10 min, flagellates were carefully transferred from the supernatant to a new tube and spun at 1500 x g for 5 min. For transmission electron microscopy, specimens were prepared following a protocol described elsewhere [29] and observed under a JEM-100CX-II microscope system. The kDNA networks were isolated by sucrose gradient ultracentrifugation using a previously described protocol [30]. The isolated kDNA were visualized on 1 % agarose gel. Endonucleases *MspI*, *MboI*, *BamHI*, *TaqI*, *HindIII*, *RsaI*, and *HaeIII* (New England Biolabs, USA) were used for restriction enzyme analysis and were carried out according to the conditions recommended by the manufacturer. Computer-simulated restriction enzyme digestion map of *T. lewisi* maxicircle was performed using the Vector NTI software suite [31].

### Deep sequencing, assembly, PCR verification

A kDNA library of *T. lewisi* was commercially prepared and 100 bp-long paired-end reads were obtained by Illumina Hiseq 2000 (Novogene, China) and assembled into contigs by Velvet version 1.2.10 software [32]. Maxicircle contigs were identified by alignment with published maxicircles of *T. cruzi* (GenBank: DQ343645), *T. rangeli* (GenBank: KJ803830.1), *T. brucei* (GenBank: M94286.1) and *L. tarentolae* (GenBank: M10126.1) using NCBI BLAST software. The assembly of contigs was confirmed and gaps between contigs (NODE_60 and NODE_165; NODE_165 and NODE_28) were filled-in by sequencing PCR products obtained with 11 pairs of primers, listed in Table 1. In order to obtain the sequence of the non-coding DR between the contigs NODE_28 and NODE_60, a forward primer adapted from the 3’ end of NODE_28 and two reverse primers from the 5’ end of NODE_60 were used for PCR amplifications. A commercial kit (Prime STAR Max DNA Polymerase, TaKaRa, China) was utilized for all PCR amplifications.

**Table 1:** Primers for PCR amplification of the *T. lewisi* maxicircle

Primer	Sequence (5’ → 3’)	Fragment position^a^
Tl Frag1-F	GCTAATTGCACTAATCGAGGT	(−552)-1446
Tl Frag1-R	GCTGGCATCCATTTCTGACT	
Tl Frag2-F	AAAGGTCCGAGCAGGTTA	977-3001
Tl Frag2-R	CTTTTCTGTGCCACGATGT	
Tl Frag3-F	ATAAGAATAAGAGGGACAAACC	2877-4831
Tl Frag3-R	CGCATCTGAACTCATAAAATAG	
Tl Frag4-F	AGGTTTTGTAGTGCGTAGTGTAC	4446-5267
Tl Frag4-R	ATTCCATTCATATTGGATAAGC	
Tl Frag5-F	TTTATTGTGAACGGTTATGCT	4851-8008
Tl Frag5-R	ACAACTTCGGATTGGACCT	
Tl Frag6-F	ATGGCTGCGAGATAAACAA	7697-9586
Tl Frag6-R	GGCATTAAAACAAAACAACTT	
Tl Frag7-F	TATTTGGATCATACGCCTTA	8980-11526
Tl Frag7-R	GGAATGATAAAGCGGGAA	
Tl Frag8-F	AAAATCCGCTAACTAAACACC	11104-12847
Tl Frag8-R	CCTAAGAAAAGGGAACTTCATAC	
Tl Frag9-F	TATTTCTAATGGGGCTTGTG	11971-13782
Tl Frag9-R	CACAGAAATCGTAATAGCAATAC	
Tl Frag10-F	GGAAGTTTACTTTTAGGAAGGC	13644-14639
Tl Frag10-R	GTGGATTCATACACCCATGAC	
Tl Frag11-F	GGAAGGACCAATCCCAGTT	14306-16095
Tl Frag11-R	TGTACGTTACAATTCGGTGTTT	
Tl DR-1 F^b^	CCATTAAAACCAAATTAGGTG	16855-(−2912)
Tl DR-1R	GGAGAGAAGGGAAAATAAGG	
Tl DR-2 F^b^	CCATTAAAACCAAATTAGGTG	16855-(−3218)
Tl DR-2R	TCGTATAAAGCGATGTGAAAG	

^a^presents fragment positions are shown relative to the start of the *12S rRNA*, and the positions located before *12S rRNA* gene are indicated with minus signs and enclosed in parentheses

^b^presents sequences of primers TlDR-1 F and TlDR-2 F are same

### Data analysis

Alignment and manual annotation of *T. lewisi* sequences were carried out by comparison with other available maxicircle sequences, including maxicircles of *T. cruzi* strain Esmeraldo (GenBank: DQ343646.1), *T. cruzi* strain marinkellei (GenBank: KC427240.1), *T. cruzi* strain Silvio (GenBank:FJ203996.1), *T. equiperdum* (GenBank: EU185800.1), *T. congolense* (Tritrypdb: T.congo_bin 13880417 to 13889953), *T. vivax* (GenBank: KM386508.1). Phylogenetic tree was constructed based on Neighbor joining or Maximum likelihood methods with 1,000 bootstrap replicates using MEGA 4.0 [33]. Dot matrix graphs of the *T. lewisi* maxicircle sequence against itself and maxicircles from other trypanosomatids were generated using EMBOSS software suite [34]. Artemis software was used to generate GC percentage graphs of the maxicircle coding region [35] and identity indices among trypanosomatid protists were calculated with BioEdit software [36]. Motifs in the DR sequence were identified and presented in LOGO diagrams by MEME software [37].

### Ethical approval

In this study, rats were treated in strict accordance to the guidelines for Medical Laboratory Animals (1998) from Ministry of Health, China, under the protocols approved by National Institute for Communicable Disease Control and Prevention and Laboratory Animal Use and Care Committee of Sun Yat-Sen University under the licenses of 2010CB53000.

## Results

### Morphology, ultrastructure, kDNA isolation and restriction enzyme digestion

**Fig. 1:**
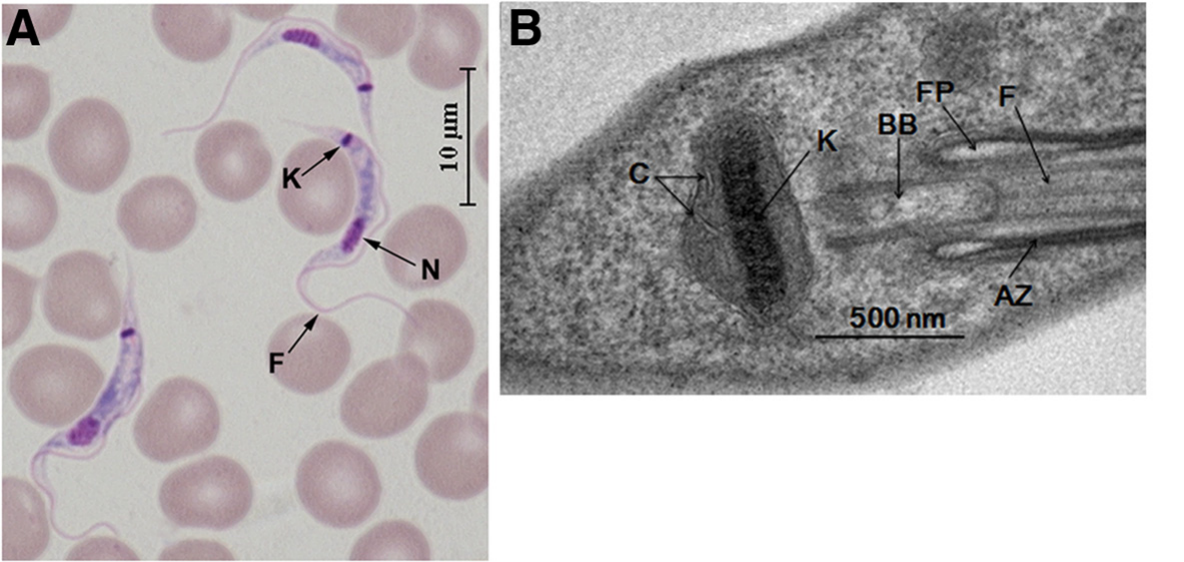
Morphology and ultrastructure of *T. lewisi*. **a** Diff Quick staining of bloodstream forms of *T. lewisi* from rat. Flagellum (F), Nucleus (N) and Kinetoplast (K) are indicated. **b** Electron micrograph of *T. lewisi* trypomastigote form. Attachment Zone (AZ), Basic Body (BB), Mitochondrial Cristae (MC), Flagellum (F), Flagellar Pocket (FP) and Kinetoplast (K) are indicated

In vertebrate blood, the predominant morphological type is the slender trypomastigote form in which a prominent kinetoplast, about one tenth of the size of the nucleus, is located close to the posterior end, as judged by Diff Quick staining (Fig. 1a). Ultrastructural analysis of this stage revealed that the kDNA disk measures 588 ± 92 nm in length and 138 ± 18 nm in width (*n* = 65) (Fig. 1b), which is similar to other trypanosomes.

A total of ~10^9^ *T. lewisi* bloodstream trypomastigotes were harvested from infected rats. High quality kDNA was obtained as judged by the 260/280 absorbance ratio of *T. lewisi* kDNA which was 1.86. To verify its quality, an aliquot of the kDNA sample was run on an agarose gel. The kDNA was intact and free from contamination of the nuclear and host DNAs, since no corresponding bands of nuclear DNA was found (See Additional file 1: Figure S1).

The restriction enzyme digestion pattern of the *T. lewisi* maxicircle was obtained by incubation with the endonucleases *MspI*, *MboI*, *BamHI*, *TaqI*, *HindIII*, *RsaI* and *HaeIII*. Following digestion, linearized fragments released from the catenated kDNA were separated by agarose gel electrophoresis and recorded (Additional file 2: Figure S2A). A high frequency of cleavages of kDNA minicircles was achieved by incubation with *MboI*, *TaqI* and *HaeIII*, as indicated by the released kDNA fragments. Most likely, all bands with molecular sizes of over 4 kb are derived from the kDNA maxicircles. The presence of some high molecular weight bands with a size greater than 20 kb, found in the samples which were digested by *MspI*, *BamHI*, *HindIII* and *HaeIII*, indicate that the full-size kDNA maxicircle is larger than 20 kb. The abundant bands smaller than 2.0 kb imply the presence of a high number of likely heterogeneous minicircles in the kDNA of *T. lewisi*. This is a good correlation with the disk-like ultrastructure of its kDNA (Fig. 1), which is clearly composed of thousands of densely packed minicircles.

### Assembly and annotation of the kDNA maxicircle

**Fig. 2:**
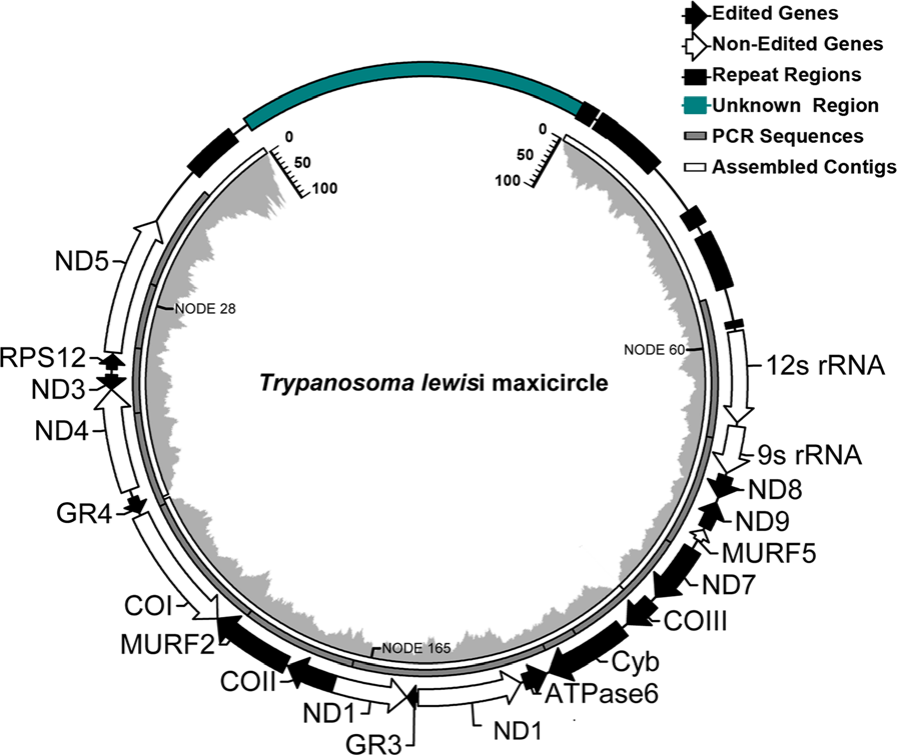
Diagram of the *T. lewisi* maxicircle*.* The diagram is composed of fourloops, from inner to outer are assembly coverage, assembled contigs, PCR sequencing and geneorganization, respectively

To obtain the full-size maxicircle sequence*,* the purified kDNA was deep sequenced on the Illumina Hiseq 2000 platform. A total of 5,742,059 raw pair-end reads were generated. After removal of adapters, poly-N reads (reads containing >10 % poly-N) and low quality reads (sQ < = 5) from raw data, we obtained 5,592,095 clean reads with Q20 (94.95 %), Q30 (87.64 %) and GC content (36.45 %). These data were finally assembled into 528 contigs using the Velvet software. As expected, no host DNA sequences were found, confirming the high purity of the sample. Three *T. lewisi* maxicircle contigs (NODE_60, NODE_165, NODE_28) were identified by the BLAST search against the *T. cruzi*, *T. rangeli*, *T. brucei* and *L. tarentolae* maxicircle sequences. The rest of the contigs are likely to be the sequences from minicircles or divergent region of maxicircle. They have an average coverage of 35x, which provides a high level of confidence for the assembly. These contigs were further verified by sequencing PCR amplicons obtained with 11 primer pairs (Additional file 3: Figure S3A), by which gaps within the coding region have been filled. A combination of three assembled contigs and 11 PCR amplicons thus produced a 20,618 bp-long sequence. The sequence covers the entire coding region (15,369 bp) and part of the non-coding region (5,249 bp) of the *T. lewisi* maxicircle, which is shown in Fig. 2.

To obtain the remaining non-coding DR sequence, two additional primer pairs (TlDR 1 F/1R and TlDR 1 F/2R) from the contigs of NODE_28 and NODE_60 were designed and repeatedly used in PCR reactions (Additional file 3: Figure S3B). However, due to numerous repetitions in this region, we were unable to assemble the whole region. Accordingly, only 3,127-bp of new sequence derived from this region was obtained. Another BLAST search using this new sequence as a query identified an additional set of contigs (NODE_805, NODE_1689, NODE_1919, NODE_587, NODE_302). Altogether, a total of 23,745 bp of the *T. lewisi* maxicircle was obtained and deposited in the GenBank (accession no. KR072974).

According to previous reports [12, 38], with the exception of some pan-edited regions, the nucleotide sequences and gene order of the maxicircle coding region are highly conserved among trypanosomatid flagellates, allowing a straightforward annotation of the *T. lewisi* kDNA maxicircle. Our data indicate that its maxicircle encodes 20 tightly clustered genes (Table 2), with a gene order syntenic with that of *T. cruzi*, *T. brucei* and *L. tarentolae*. We have also launched a computer-simulated virtual restriction digestion and electrophoresis (VRDE) to evaluate the physical map of this assembled maxicircle (Additional file 2: Figure S2B). Since VRDE considers only complete cleavage by restriction enzymes, it predicted at least 42 restriction sites for *RsaI* and *TaqI*, butonly several target sites for *HindIII*, *HaeIII* and *MspI*, and just one for *BamHI*. All computer-simulated restriction fragments which were greater than 4.0 kb from the 23,745 bp-long sequence were experimentally verified by restriction digests (asterisks in Additional file 2: Figure S2B), confirming a high correlation between both approaches. However, a handful of weak bands appeared in agarose gels that were not predicted by the software (question marks in Additional file 2: Figure S2B) implying either that they originate from an unknown region or from an incomplete digestion.

**Table 2:** Gene positions and lengths in the *T. lewisi* maxicircle

Gene	RNA editing	*T. lewisi* position	*T. lewisi* length	*T. cruzi* length^d^	*T. brucei* length^e^	*L. tarentolae* length^e^
*12S rRNA*	/	1–1168	1168	1161	1149	1173
*9S rRNA*	/	1218–1825	608	608	611	611
*ND8*	Extensive	1865–2149	285	279	266	266
*DN9* ^a^	Extensive	2218–2567	350	338	321	291
*MURF5* ^a^	None	2581–2821	241	264	234	303
*ND7* ^b^	Extensive	2887–3657	771	755	702	1144
*COIII* ^b^	Extensive	3741–4154	414	424	439	852
*Cyb*	Minor	4242–5321	1080	1080	1080	1079
*ATPase6* ^b^	Extensive	5359–5662	304	336	369	603
*MURF1/*(*ND2*)^a^	None	5704–7044	1341	1341	1237	1332
*GR3* ^c^	Extensive	7033–7155	~123	~119	~164	167
*ND1* ^a^	None	7172–8113	942	942	957	942
*COII*	Minor	8115–8743	629	629	626	629
*MURF2*	Minor	8770–9822	1053	1056	1041	1045
*COI* ^a^	None	9813–11462	1650	1650	1734	1650
*GR4* ^a^	Extensive	11508–11719	212	207	185	189
*ND4*	None	11824–13137	1314	1314	1311	1314
*ND3* ^a^	Extensive	13126–13312	187	193	256	187
*RPS12*	Extensive	13383–13572	190	191	172	182
*ND5*	None	13597–15369	1773	1770	1770	1773

Gene positions are shown relative to the start of the gene *12S rRNA*

^a^presents those genes that are encoded by the reverse strand

^b^presents those genes that are 5’-edited in *L. tarentolae*

^c^presents the two end positions of *GR3* from *T. lewisi*, *T. cruzi* and *T. brucei* are uncertain

^d^presents the maxicircle gene lengths of *T. cruzi* that are cited from the paper of Ruvalcaba-Trejo [16]

^e^presents calculations of the maxicircle gene lengths of *T. brucei* and *L. tarentolae* that are based on the data from GenBank (M94286.1 and M10126.1, respectively)

### Comparative analysis of trypanosomatid maxicircles

A dot matrix analysis was employed to compare the *T. lewisi* maxicircle with those of *T. cruzi, T. brucei, T. rangeli* and *L. tarentolae* (Additional file 4: Figure S4). Similar sequences between two species are indicated by diagonal lines, each dot represents a full identity over a 10 bp-long window. Based on the graphs, we can clearly see that the *T. lewisi* maxicircle displays higher overall sequence identity to *T. cruzi, T. rangeli* and *T. brucei* than to *L. tarentolae*. The sequences of coding regions and especially the region corresponding to the genes the transcripts of which do not undergo RNA editing (*12S rRNA*, *9S rRNA*, *ND2*, *ND1, COI*, *ND4* and *ND5*) have a high degree of identity among all compared species, while much bigger differences were found in their Divergent Region (DR) sequences. Variations in the coding regions which caused shifts and breaks on the diagonal line occurred mainly in genes with transcripts subject to (extensive) editing, namely *ND8*, *ND9*, *ND7*, *COIII*, *ATPase6* and *GR4*. An array of substantial breaks appeared in the comparison with *L. tarentolae* (Additional file 4: Figure S4D, red box). RNA editing of *ND7*, *COIII* and *ATPase6* in *L. tarentolae* were shown to be limited to the 5’ region but are extensive in the same mRNAs in *T. brucei* [39, 40]. These breaks suggest that the editing patterns in *T. lewisi* may be more similar to other trypanosomes than to *L. tarentolae*. Moreover, the analysis of the maxicircle shows that it also carries guide RNA genes. This is judged by a guide RNA bearing information compatible with the editing of the *COII* mRNA. However, without validation at the RNA level, these guide RNAs have not yet been included in this annotation.

### GC plot and comparative analysis of maxicircle coding regions and mature mRNA predictions

The dot matrix comparison analysis mentioned above indicated that editing pattern in the *T. lewisi* maxicircle may be similar to those in *T. cruzi* and *T. brucei*. In order to further predict editing sites, we analyzed the GC content of the *T. brucei* maxicircle (Additional file 5: Figure S5A), as a reference whose RNA editing pattern is well documented, and the GC content of the *T. lewisi* maxicircle coding regions. Indeed, the corresponding regions of extensively edited *T. brucei* genes (*ND8, ND9, ND7, COIII, ATPase6, GR3, GR4, ND3* and *RPS12*) have higher GC contents than the non-edited genes. Since the GC scatter plot of *T. lewisi* is similar to that of *T. brucei* (Additional file 5: Figure S5A and S5B), high GC content genes may produce pan-edited transcripts, while those of *COII*, *Cyb* and *MURF2* seem to undergo only limited editing. The continuous open reading frames of the remaining 8 genes testify to the lack of editing.

Pairwise alignment was done to evaluate the level of identity in the entire coding sequences and the edited and non-edited genes (both nucleotide and amino acid sequences) between *T. lewisi* and four trypanosomatids (Table 3). The entire coding sequence of *T. lewisi* shows highest and lowest levels of identity to *T. cruzi* and *L. tarentolae*, respectively*.* Sequences not subject to editing exhibit a higher level of identity among species than the extensively edited ones. We would like to point out that high identity among species was not confined to the non-edited genes, but was also observed in the 5’ edited genes, indicating the presence of conserved editing patterns in these genes.

**Table 3:** Average percentage identity among the maxicircle DNA from five trypanosomatid species

Comparison of *T. lewisi*	Entire coding region	5’-editedgenes	Extensively-edited genes	rRNAs	Non-edited genes	AAs of Non-edited genes
vs. *T. cruzi*	78.0 %	82.0 %	74.2 %	83.5 %	79.8 %	82.7 %
vs. *T. rangeli*	77.2 %	82.8 %	73.8 %	84.5 %	79.3%^a^	82.5%^a^
vs. *T. brucei*	74.1 %	81.8 %	61.7 %	79.4 %	77.2 %	78.4 %
vs. *L. tarentolae*	66.4 %	77.6 %	50.8 %^b^	78.4 %	75.5 %	74.4 %

Entire coding region: starting from the 5’end of *12S rRNA* to the 3’ end of *ND5*

5’-edited genes: *Cyb*, *COII*, *MURF2*

Extensively-edited genes: *ND9*, *ND8*, *ND7*, *COIII*, *ATPase6*, *GR3*, *GR4*, *RPS12*

Non-edited genes: *MURF2*/(*ND2*), *ND1*, *COI*, *ND4*, *ND5*

Proteins: *MURF* (*ND2*), *ND1*, *ND4*, *ND5*, *COI*

^a^presents gene *ND1* was not included in the analysis of non-edited genes and proteins of *T. rangeli* due to the lack of complete sequence of this species

^b^presents gene *COIII, ND7* and *ATPase6* were not included in the analysis in *L. tarentolae* due to different RNA editing patterns

### Sequence analysis of maxicircle divergent region

**Fig. 3:**
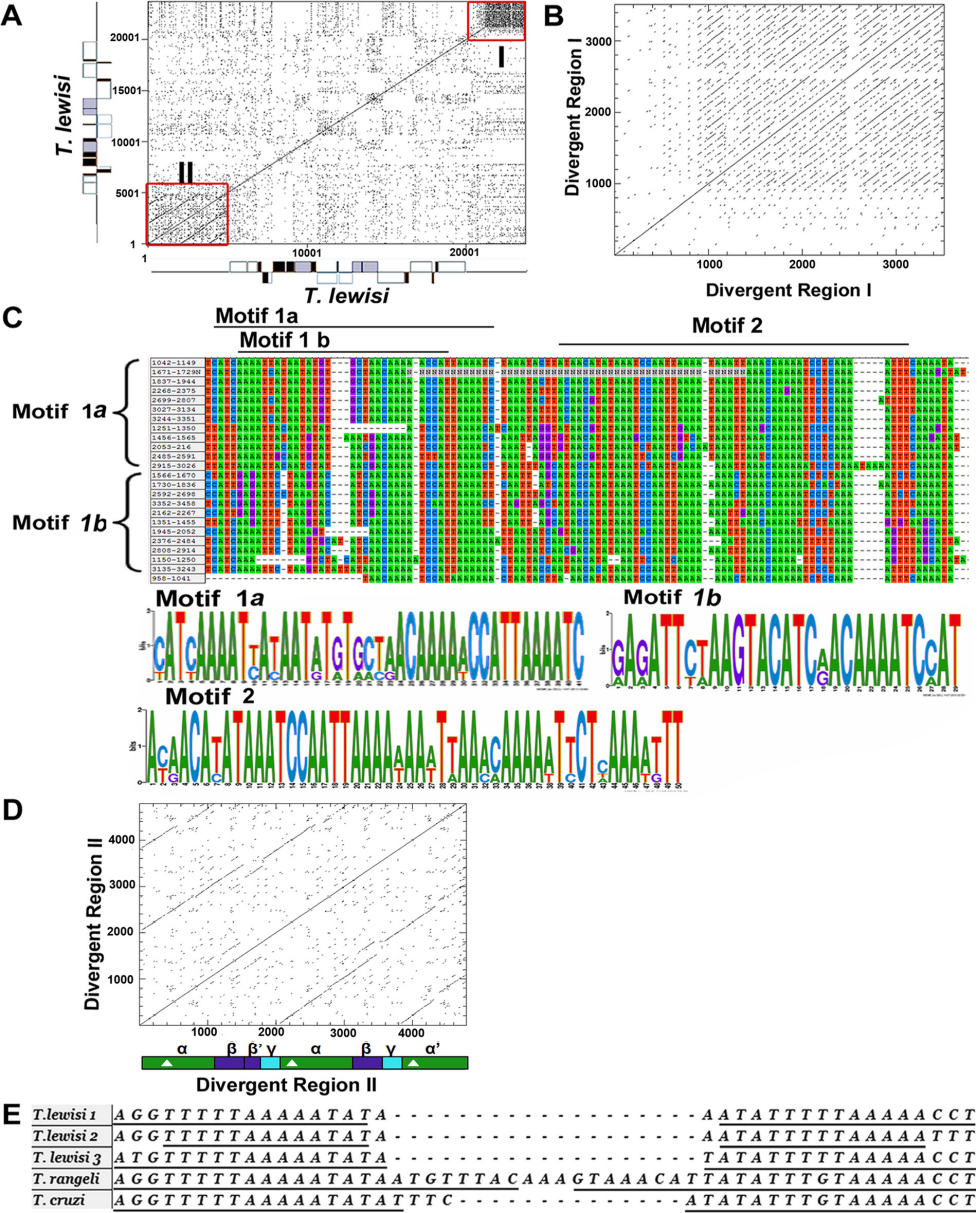
Sequence analyses of the *T. lewisi* maxicircle divergent region. Self Dottup graph of the *T. lewisi* entire maxicircle (**a**), DR section I (**b**), DR section II (**d**). Each dot represents an exact match over of 10 nt. Two distinct sections of DR region (I and II) are indicated in (**a**) with red boxes. The repeated elements (α, β, γ, α’ and β’) identified from Dottup graph in (**d**) are illustrated, three palindromes within elements are indicated with white triangles. **c** The repetitive sequences from the section I were aligned and the position of three motifs is indicated with black line. LOGO diagrams show nucleotides at a given position of each motif and their relative frequency indicated by height. **e** The palindrome sequences from the DR of *T. lewisi*, *T. rangeli* and *T. cruzi* are shown with the inverted repeats underlined

A common theme of the maxicircle DRs is the presence of various repeat arrays, which is also the case for *T. lewisi*. Mutual maxicircle comparison of data from the dot matrix identified two distinct sections (I, II) in the DR, flanking either *12S rRNA* or *ND5* (Fig. 3a). Section I is composed of short and highly repetitive units of about 100 bp (Fig. 3b), while section II consists of several large duplications (Fig. 3d). Three motifs (motif 1a, motif 1b and motif 2) were found in section I by MEME analysis (Fig. 3c). Although they showed no similarity with other trypanosomatid species, the high confidence E-value (all < 1.0e-016) of these motifs indicate their functionality, particularly for A_5_C-element-containing motif 1a. Detailed analysis recognized that section II has a series of tandem elements, namely α, β, and their shorter version α’ and β’, and non-repeated element γ (Fig. 3d). Three AT-rich palindromes are present in the element α/α’. In the first element α, palindrome 1 is 34 bp long (AGGTTTTTAAAAATATAAATATTTTTAAAAACCT), located 4,666 nt upstream of *12SrRNA*. In the second element α, palindrome 2 is 28 bp long (TTTTTAAAAATATAAATATTTTTAAAAA) and 2,652 nt upstream of *12SrRNA*. Although the third element α was labeled as α’ due to limited deletions, it still contains a 34 bp long palindrome (ATGTTTTTAAAAATATA-TATATTTTTAAAAACCT), 879 nt upstream of the same gene. Comparison of these three palindromes with the maxicircles of *T. cruzi* and *T. rangeli* enabled the identification of similar palindromes in these species (Fig. 3e). Conservation of these AT-rich palindromes testifies to their functionality.

### Maxicircle-base phylogenetic inference

**Fig. 4:**
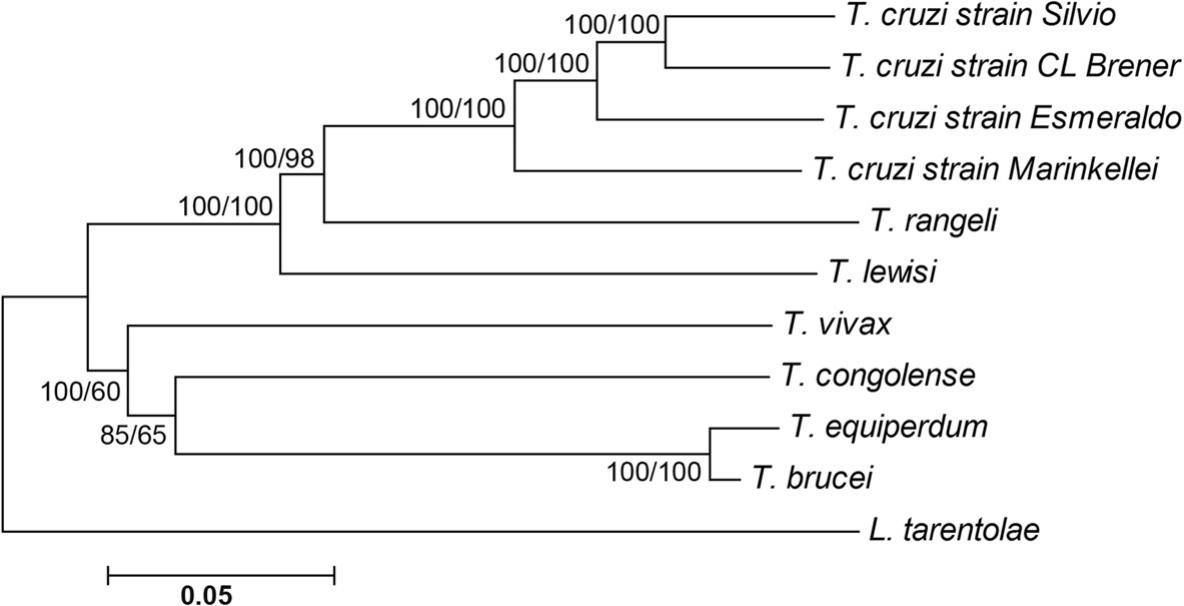
Phylogenetic tree of maxicircle coding sequences from trypanosomatid species. Phylogenetic tree is performed based on Neighbor joining or Maximum likelihood methods with 1,000 bootstrap replicates. The scale bar represents the number of nucleotide substitutions per site. Partial *T. congolense* (9 kb) was retrieved from Tritrypdb by BLAST using other trypanosome maxicircle sequences

Mitochondrial DNA sequences are considered valuable markers for the inference of phylogenetic relationships [24]. Use of the entire maxicircle coding sequence would be superior to single gene-based phylogenies. To further investigate the genetic relationships among five trypanosomatid species, their maxicircle coding regions were aligned and used to build a neighbor-joining tree, which revealed that *T. lewisi* clusters with *T. cruzi* and *T. rangeli* with 100 % confidence (Fig. 4). Although both *T. rangeli* and *T. lewisi* belong to the same subgenus *Herpetosoma*, *T. rangeli* is more closely related, with over 95 % confidence, to *T. cruzi* than to *T. lewisi*. African trypanosomes *T. brucei*, *T. congolense*, *T.vivax* and *T. equiperdum* were present in the second clade and all belong to the salivarian subgroup. Not surprisingly, *L. tarentolae* is separated from *T. lewisi* by a large genetic distance.

## Discussion

Trypanosomatid protists including the pathogens causing leishmaniasis and trypanosomiasis in animals and humans invariably possess the uniquely structured kDNA [10, 41]. Since *T. lewisi* was recently defined as an opportunistic zoonotic and human blood parasite [5], it became particularly relevant to investigate its molecular and cellular features. Here we undertook an in-depth analysis of its maxicircle kDNA and compared it with its homologues in other trypanosomatids. We have combined deep sequencing and PCR in order to assemble, with sufficient sequence coverage, a 23.7 kb-long region of the *T. lewisi* maxicircle. The estimated size of the complete maxicircle is approximately 24 kb, which is within the size range of 20 to 40 kb, estimated for other trypanosomatids studies thus far [10, 11].

The uridine insertion and/or deletion type of RNA editing is the most characteristic feature of the kinetoplastid mitochondrion. It is required to remove multiple frame shifts from about a dozen of transcripts, which encode subunits of respiratory complexes, rendering this post-transcriptional machinery essential [20, 22, 42, 43]. Interestingly, both the set of transcripts and the extent of their editing are species-specific. For example, substantial differences in the editing patterns of *ND7*, *COIII* and *ATPase6* have been documented; they are extensively edited in *T. brucei*, while only limited editing of the same transcripts occurs in *L. tarentolae*. Consequently, these so-called cryptogenes are much shorter in *T. brucei* [39]. Based both on the similarity of pre-edited gene sequences and the GC content plot of coding region, *T. lewisi* seems to have the *T. cruzi*–like editing pattern*.* However, this preliminary conclusion will have to be tested at the RNA level.

Even bigger inter-specific and inter-strain differences have been described for the maxicircle non-coding DR [38, 44, 45]. Its common feature is the presence of various repetitive sequences, which are also present in the DR of *T. lewisi*. Due to the absence of larger ORFs, it was proposed that this region may play a structural role and is also required for replication. This notion is based on the presence of a 12 bp-long conserved sequence block (CSB) in the DRs of *T. brucei* [46], *Crithidia oncopelti* [47] *Leptomonas collosoma a*nd *Leptomonas seymouri* [48]. CSB is a highly conserved replication origin of the kDNA minicircles, and it may fulfill the same role also in the maxicircles [14, 46]. We attribute our failure to find CSB to its presence in the missing piece of the maxicircle DR of *T. lewisi*.

Furthermore, two palindromic elements were reported from the DRs, namely 39 bp and about 40 bp-long palindromes in *T. cruzi* and *Leishmania* spp., respectively [38, 48]. Although, these two palindromes were divergent in sequence, they both contain an A_5_C-element, which reflects their putative association with transcription initiation [49] or transcription factor binding sites [48]. In addition, palindromes may serve in a range of molecular capacities, such as the recognition sequences for restriction enzymes [50], binding sites for DNA-binding proteins [51] or may participate in control of gene expression [52]. As expected, we have identified three palindromes in the DR of *T. lewisi*, which are all homologous to the palindrome of *T. cruzi*. It is worth noting that we have also found a similar 53 bp-long palindrome sequence in the maxicircle DR of *T. rangeli*. All of these palindromes consist of the A_5_C element and two T_5_A_5_ elements, implying these palindromes may play a significant role, yet their function remains unknown. Further functional research in *T. lewisi* and other kinetoplastids will be required to establish the role of these detailed sequence elements in the function and evolution of kinetoplasts and their DNA.

Mutual comparison with four members of the genus *Trypanosoma* and *L. tarentolae* using dot matrix comparison and average percentage identities revealed a high level of sequence similarity, with *T. cruzi* being more closely related. Our phylogenetic analysis also confirmed the well-established close relatedness of *T. cruzi* and *T. lewisi*. It is interesting to note that *T. rangeli* and *T. lewisi* are morphologically similar and both have been classified into the subgenus of *Herpetosoma*, while *T. cruzi* is affiliated with the subgenus *Schizotrypanum* [2]. However, *T. rangeli* is known to be quite different from others members of the genus *Herpetosoma*, both in terms of pathogenicity and transmission pathways, as well as based on the internal transcribed spacer and SSU rRNA sequences [7, 53, 54]. Hence, its reclassification into another subgenus has been suggested [53, 54], and our data from the mitochondrial genome lend further support for such a move*.*

The kinetoplast plays an important role in the life cycle of trypanosomes and, in particular, with respect to maintaining vector borne transmission cycles. A wide range of trypanosome species are found in an equally wide range of wild animal hosts globally. Typically, species of trypanosomes tend to have specific host/vector systems [2], such as the rat/rat flea with *T. lewisi*, discussed here, or other examples like the badger/badger flea in the case of *T. pestanai* [55, 56]. Loss of parts of the kDNA have been demonstrated in trypanosome species, such as *T. evansi* and *T. equiperdum*, that may have escaped their traditional host vector systems [27]. These species are suggested to be only a few genetic steps away from the important human pathogen, *T. brucei* [28]. The discovery of human infections caused by atypical trypanosomes, such as *T. evansi*, *T. congolense a*nd *T. lewisi* [3, 8], raises concerns about the potential for human infectivity of many other trypanosomes with sylvatic cycles. Some trypanosome species, such as *T. evansi* and *T. equiperdum*, have undergone an evolutionary adaptation to mechanical transmission between their specific hosts (e.g. camels and horses, respectively) without the need for developmental stages in a vector. The lack of kDNA maxicircles, found in *T. evansi* strains examined, is linked to a failure of key differentiation processes in *T. brucei*, which have been considered as an example of a cancer of parasitic protozoa [57]. More research is required to understand the role of kDNA in the evolution of diversity, transmission and pathogenicity within the kinetoplastids.

## Conclusions

This is the first detailed analysis of the *T. lewisi* maxicircle. We show that it has a high level of similarity and the same gene order as other trypanosomatids. The most related species is, somewhat unexpectedly, *T. cruzi*. The predicted pattern of RNA editing is also quite similar to *T. cruzi.* A duplicated A_5_C element-containing palindrome was found in the DR of *T. lewisi*, suggesting its functional conservation*.* The sequence obtained from this neglected human pathogen provides information suitable for generation of a diagnostic assay.

## Additional files

Additional file 1: Figure S1. Genome DNA and kinetoplast DNA preparations from *T. lewisi* were resolved on a 1% agarose gel. Genome DNA and kinetoplast DNA preparations from *T. lewisi* were resolved on a 1% agarose gel. Lanes: M, Molecular marker DL10000 (TaKaRa, Dalian, China); gDNA, genome DNA; kDNA, kinetoplast DNA. (PDF 88 kb) Additional file 2: Figure S2. Restriction endonuclease analysis of *T. lewisi* kDNA. A) Ethidium Bromide-stained agarose gel showing restriction endonuclease digestion of *T. lewisi* kDNA with seven restriction enzymes. Marker, DL10000 (TaKaRa, Dalian, China). Note the presence of the kDNA network in some slots, indicating integrity of its network. B) Computer-simulated virtual restriction patterns derived from the 23745 bp *T. lewisi* maxicircle with the same set of restriction sites from (A). The larger, >4.0 kb-long fragments which were identified in (A), were marked with asterisks. A few weak bands over 4.0 kb in length in (A), which were not predicted by the software, were labeled with question marks. (PDF 144 kb) Additional file 3: Figure S3. PCR amplification of *T. lewisi* maxicircles. A) Verification of the maxicircle coding region by PCR amplification using 11 pairs of primers, as visualized on a 1.0 % agarose gel. Marker, DL10000 (TaKaRa, Dalian, China). B) A 1.0 % agarose gel displaying PCR amplicons from the maxicircle divergent region obtained with two primer pairs. The 1F/1R, amplicon with TlDR 1F/1R primers, and the 1F/2R, amplicon with TlDR 1F/2R primers. Marker, 1 kb DNA Ladder (TIANGEN, China) and DL10000 (TaKaRa, Dalian, China). (PDF 142 kb) Additional file 4: Figure S4. Dottup plot comparative analysis of maxicircle sequence of *T. lewisi* against maxicircle sequences of *T. cruzi* (A)*, T. rangeli* (B)*, T. brucei* (C) and *L. tarentolae* (D), respectively. Diagonal lines indicate that the DNA sequences of two compared species are identical in the corresponding regions. Each dot represents an exact match over of 10 nt. A remarkable break region in *T. lewisi* vs. *L. tarentolae* is indicated by a red box. (PDF 339 kb) Additional file 5: Figure S5. Graphs show the GC percentage of *T. brucei* (A) and *T. lewisi* (B) maxicircle coding regions. The regions where the percentage GC content value lies above the dashed lines are likely to undergo RNA editing. The window size used for this analysis was100 nt. (PDF 193 kb)

## Abbreviations

CO: Cytochrome oxidase
CSB: Conserved sequence block
GR: G-rich region
Cyb: Apocytochrome b
DR: Divergent region
gRNA: Guide RNA
ITS: Internal transcribed spacer
kDNA: Kinetoplast DNA
MURF: Mitochondrial unidentified reading frame
ND: NADH dehydrogenase
ORF: Open reading frame
RPS12: Ribosomal protein S12
