# Erratum to: Analysis of the mitochondrial maxicircle of *Trypanosoma lewisi*, a neglected human pathogen

**DOI:** 10.1186/s13071-016-1297-8

**Published:** 2016-01-13

**Authors:** Ruo-Hong Lin, De-Hua Lai, Ling-Ling Zheng, Jie Wu, Julius Lukeš, Geoff Hide, Zhao-Rong Lun

**Affiliations:** Center for Parasitic Organisms, State Key Laboratory of Biocontrol, School of Life Sciences and Key Laboratory of Tropical Diseases and Control of the Ministry of Education, Zhongshan School of Medicine, Sun Yat-Sen University, Guangzhou, 510275 The People’s Republic of China; State Key Laboratory of Biocontrol and Key Laboratory of Gene Engineering of the Ministry of Education, School of Life Sciences, Sun Yat-Sen University, Guangzhou, 510275 The People’s Republic of China; Institute of Parasitology, Biology Centre, Czech Academy of Sciences and Faculty of Science, University of South Bohemia, České Budějovice, Czech Republic; Canadian Institute for Advanced Research, Toronto, Canada; Ecosystems and Environment Research Centre and Biomedical Research Centre, School of Environment and Life Sciences, University of Salford, Salford, UK

Unfortunately, after the publication of our work [1], we noticed a typo and a mistake in Fig. 2. In Fig. 2, two genes were both labelled with the same abbreviation (*ND1*); these should have been labelled as *ND1* (between COII and GR3) and *ND2* (between GR3 and ATPase6). In addition, the orientation of ATPase6 should have been the same as Cyb.

**Fig. 2:**
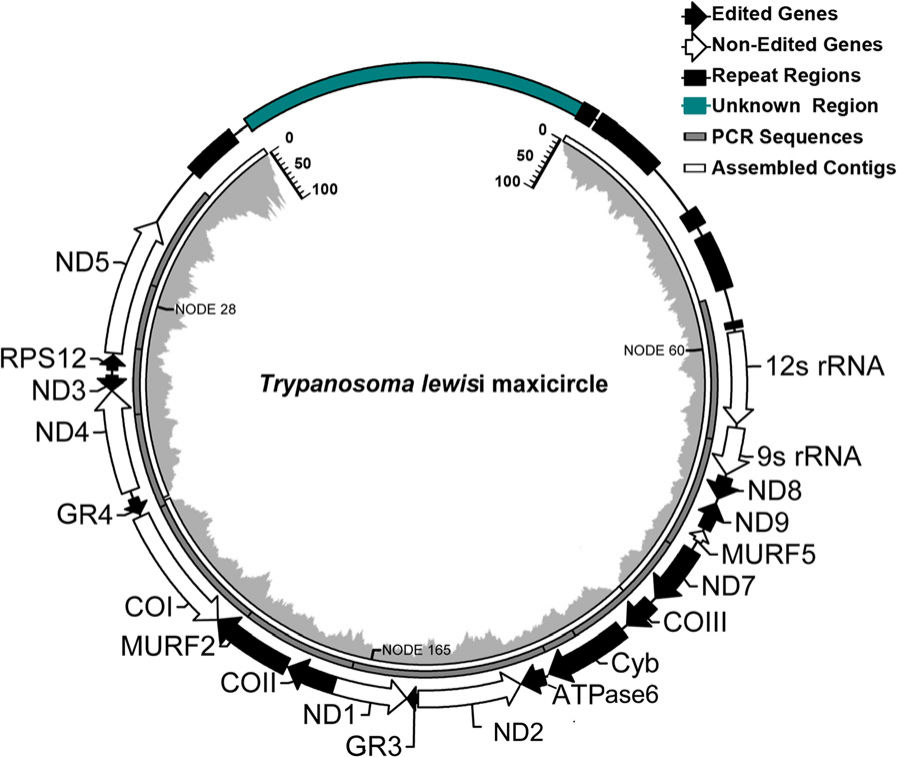
Diagram of the *T. lewisi* maxicircle*.* The diagram is composed of four loops, from inner to outer are assembly coverage, assembled contigs, PCR sequencing and gene organization, respectively

A corrected version of Fig. 2 is included below.
