# Supplementary material for: Analysis of the mitochondrial maxicircle of Trypanosoma lewisi, a neglected human pathogen
Source: Parasit Vectors. 2015 Dec 30;8:665. doi: 10.1186/s13071-015-1281-8 (PMC4696184; doi:10.1186/s13071-015-1281-8)
Supplement: Additional file 1: Figure S1. — Genome DNA and kinetoplast DNA preparations from T. lewisi were resolved on a 1% agarose gel. Genome DNA and kinetoplast DNA preparations from T. lewisi were resolved on a 1% agarose gel. Lanes: M, Molecular marker DL10000 (TaKaRa, Dalian, China); gDNA, genome DNA; kDNA, kinetoplast DNA. (PDF 88 kb) [file 13071_2015_1281_MOESM1_ESM.pdf]

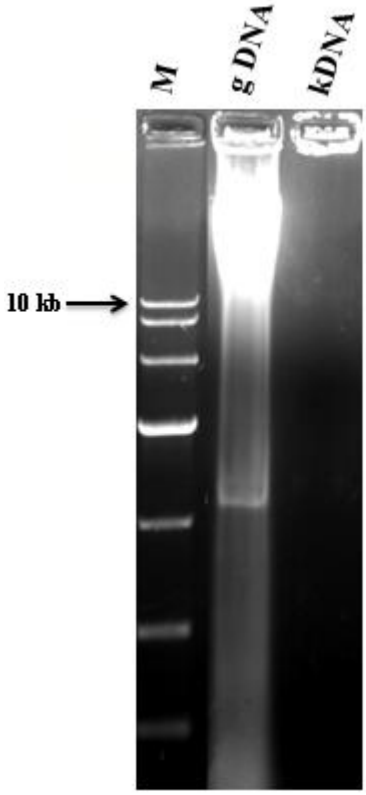

Additional file 1: Figure S1.
Genome DNA and kinetoplast DNA preparations from *T. lewisi* were resolved on a 1% agarose gel. Lanes: M, Molecular marker DL10000 (TaKaRa, Dalian, China); gDNA, genome DNA; kDNA, kinetoplast DNA.
