# Supplementary material for: Analysis of the mitochondrial maxicircle of Trypanosoma lewisi, a neglected human pathogen
Source: Parasit Vectors. 2015 Dec 30;8:665. doi: 10.1186/s13071-015-1281-8 (PMC4696184; doi:10.1186/s13071-015-1281-8)
Supplement: Additional file 2: Figure S2. — Restriction endonuclease analysis of T. lewisi kDNA. A) Ethidium Bromide-stained agarose gel showing restriction endonuclease digestion of T. lewisi kDNA with seven restriction enzymes. Marker, DL10000 (TaKaRa, Dalian, China). Note the presence of the kDNA network in some slots, indicating integrity of its network. B) Computer-simulated virtual restriction patterns derived from the 23745 bp T. lewisi maxicircle with the same set of restriction sites from (A). The larger, >4.0 kb-long fragments which were identified in (A), were marked with asterisks. A few weak bands over 4.0 kb in length in (A), which were not predicted by the software, were labeled with question marks. (PDF 144 kb) [file 13071_2015_1281_MOESM2_ESM.pdf]

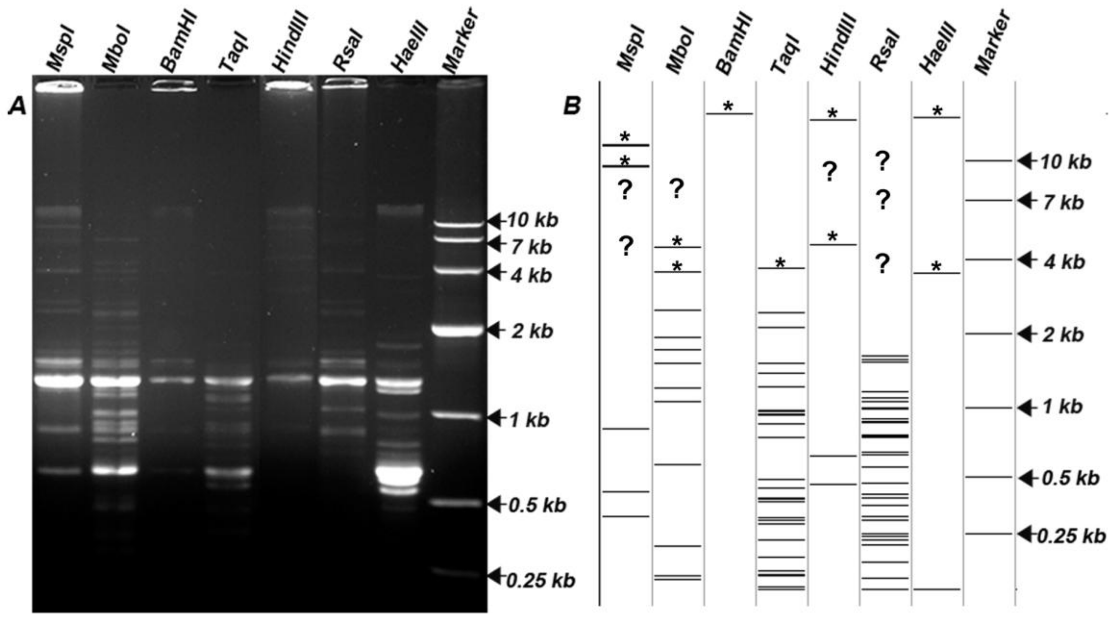

Additional file 2: Figure S2.

Restriction endonuclease analysis of *T. lewisi* kDNA. A) Ethidium Bromide-stained agarose gel showing restriction endonuclease digestion of *T. lewisi* kDNA with seven restriction enzymes. Marker, DL10000 (TaKaRa, Dalian, China). Note the presence of the kDNA network in some slots, indicating integrity of its network. **B)** Computer-simulated virtual restriction patterns derived from the 23745 bp *T. lewisi* maxicircle with the same set of restriction sites from (A). The larger, >4.0 kb-long fragments which were identified in (A), were marked with asterisks. A few weak bands over 4.0 kb in length in (A), which were not predicted by the software, were labeled with question marks.
