# Supplementary material for: Analysis of the mitochondrial maxicircle of Trypanosoma lewisi, a neglected human pathogen
Source: Parasit Vectors. 2015 Dec 30;8:665. doi: 10.1186/s13071-015-1281-8 (PMC4696184; doi:10.1186/s13071-015-1281-8)
Supplement: Additional file 3: Figure S3. — PCR amplification of T. lewisi maxicircles. A) Verification of the maxicircle coding region by PCR amplification using 11 pairs of primers, as visualized on a 1.0 % agarose gel. Marker, DL10000 (TaKaRa, Dalian, China). B) A 1.0 % agarose gel displaying PCR amplicons from the maxicircle divergent region obtained with two primer pairs. The 1F/1R, amplicon with TlDR 1F/1R primers, and the 1F/2R, amplicon with TlDR 1F/2R primers. Marker, 1 kb DNA Ladder (TIANGEN, China) and DL10000 (TaKaRa, Dalian, China). (PDF 142 kb) [file 13071_2015_1281_MOESM3_ESM.pdf]

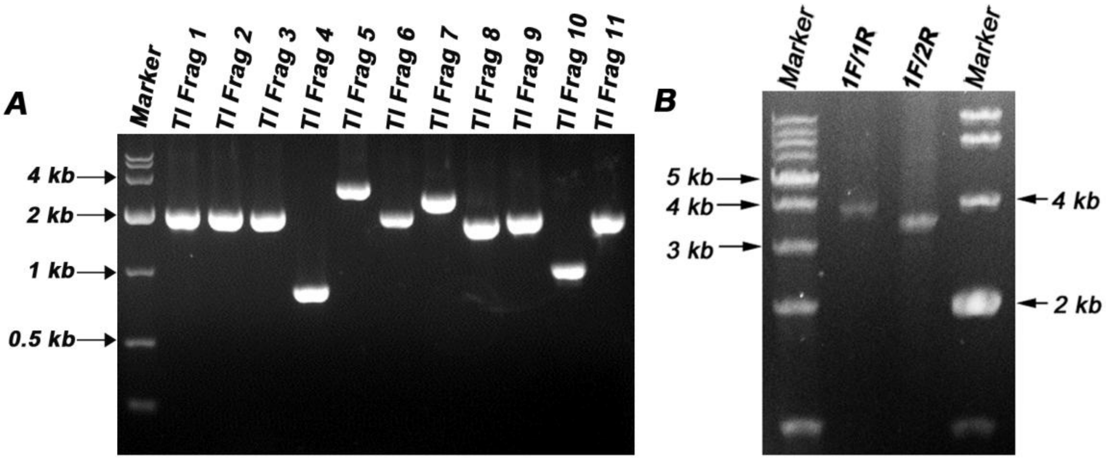

Additional file 3: Figure S3.

PCR amplification of *T. lewisi* maxicircles. A) Verification of the maxicircle coding region by PCR amplification using 11 pairs of primers, as visualized on a 1.0 % agarose gel. Marker, DL10000 (TaKaRa, Dalian, China). **B)** A 1.0 % agarose gel displaying PCR amplicons from the maxicircle divergent region obtained with two primer pairs. The 1F/1R, amplicon with TlDR 1F/1R primers and the1F/2R, amplicon with TlDR 1F/2R primers. Marker, 1 kb DNA Ladder (TIANGEN, China) and DL10000 (TaKaRa, Dalian, China).
