# Supplementary material for: Analysis of the mitochondrial maxicircle of Trypanosoma lewisi, a neglected human pathogen
Source: Parasit Vectors. 2015 Dec 30;8:665. doi: 10.1186/s13071-015-1281-8 (PMC4696184; doi:10.1186/s13071-015-1281-8)
Supplement: Additional file 4: Figure S4. — Dottup plot comparative analysis of maxicircle sequence of T. lewisi against maxicircle sequences of T. cruzi (A), T. rangeli (B), T. brucei (C) and L. tarentolae (D), respectively. Diagonal lines indicate that the DNA sequences of two compared species are identical in the corresponding regions. Each dot represents an exact match over of 10 nt. A remarkable break region in T. lewisi vs. L. tarentolae is indicated by a red box. (PDF 339 kb) [file 13071_2015_1281_MOESM4_ESM.pdf]

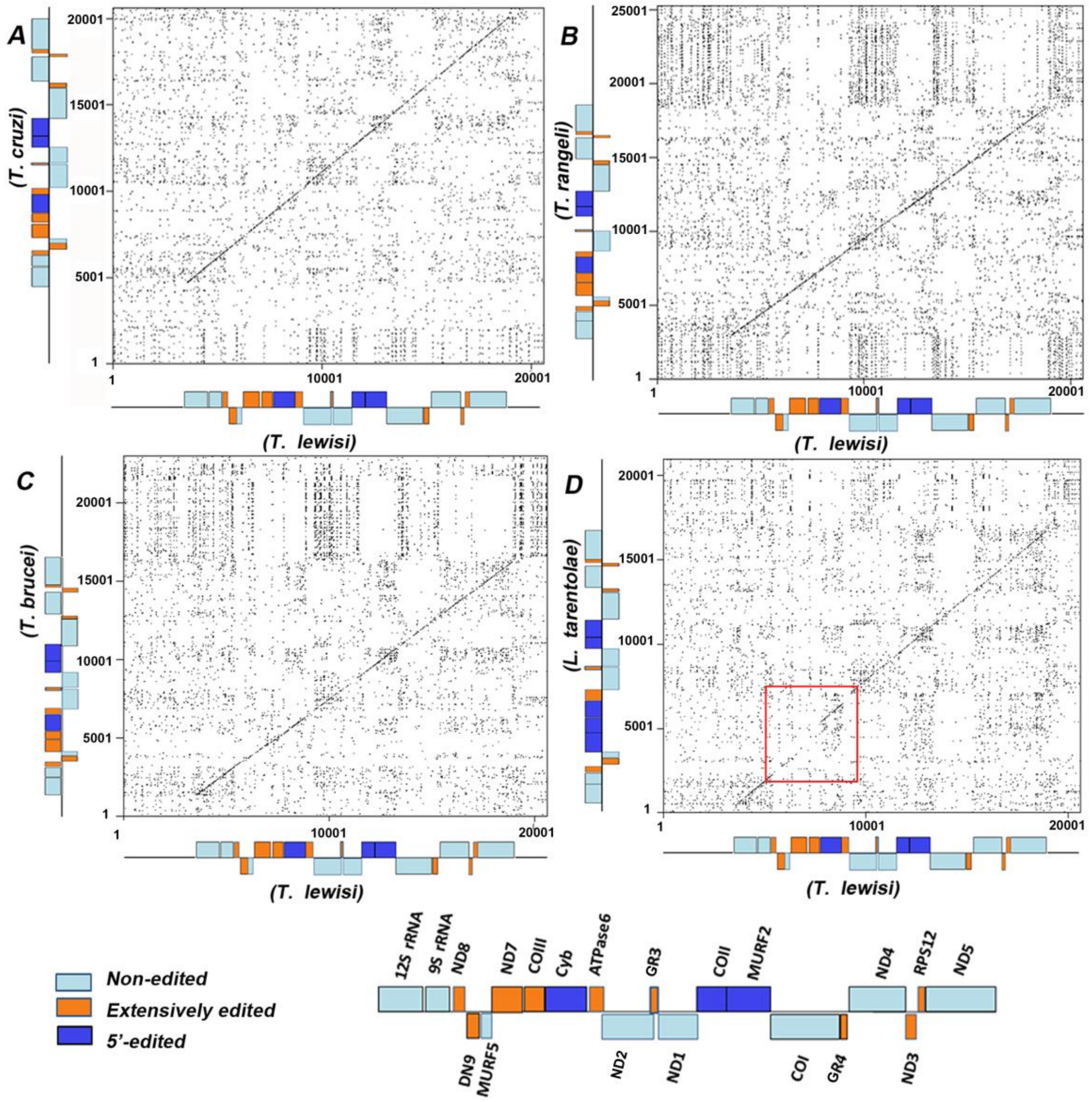

Additional file 4: Figure S4.

Dottup plot comparative analysis of maxicircle sequence of *T. lewisi* against maxicircle sequences of *T. cruzi* (A), *T. rangeli* (B), *T. brucei* (C) and *L. tarentolae* (D), respectively. Diagonal lines indicate that the DNA sequences of two compared species are identical in the corresponding regions. Each dot represents an exact match over of 10 nt. A remarkable break region in *T. lewisi* vs. *L. tarentolae* is indicated by a red box.
