# Supplementary material for: Analysis of the mitochondrial maxicircle of Trypanosoma lewisi, a neglected human pathogen
Source: Parasit Vectors. 2015 Dec 30;8:665. doi: 10.1186/s13071-015-1281-8 (PMC4696184; doi:10.1186/s13071-015-1281-8)
Supplement: Additional file 5: Figure S5. — Graphs show the GC percentage of T. brucei (A) and T. lewisi (B) maxicircle coding regions. The regions where the percentage GC content value lies above the dashed lines are likely to undergo RNA editing. The window size used for this analysis was100 nt. (PDF 193 kb) [file 13071_2015_1281_MOESM5_ESM.pdf]

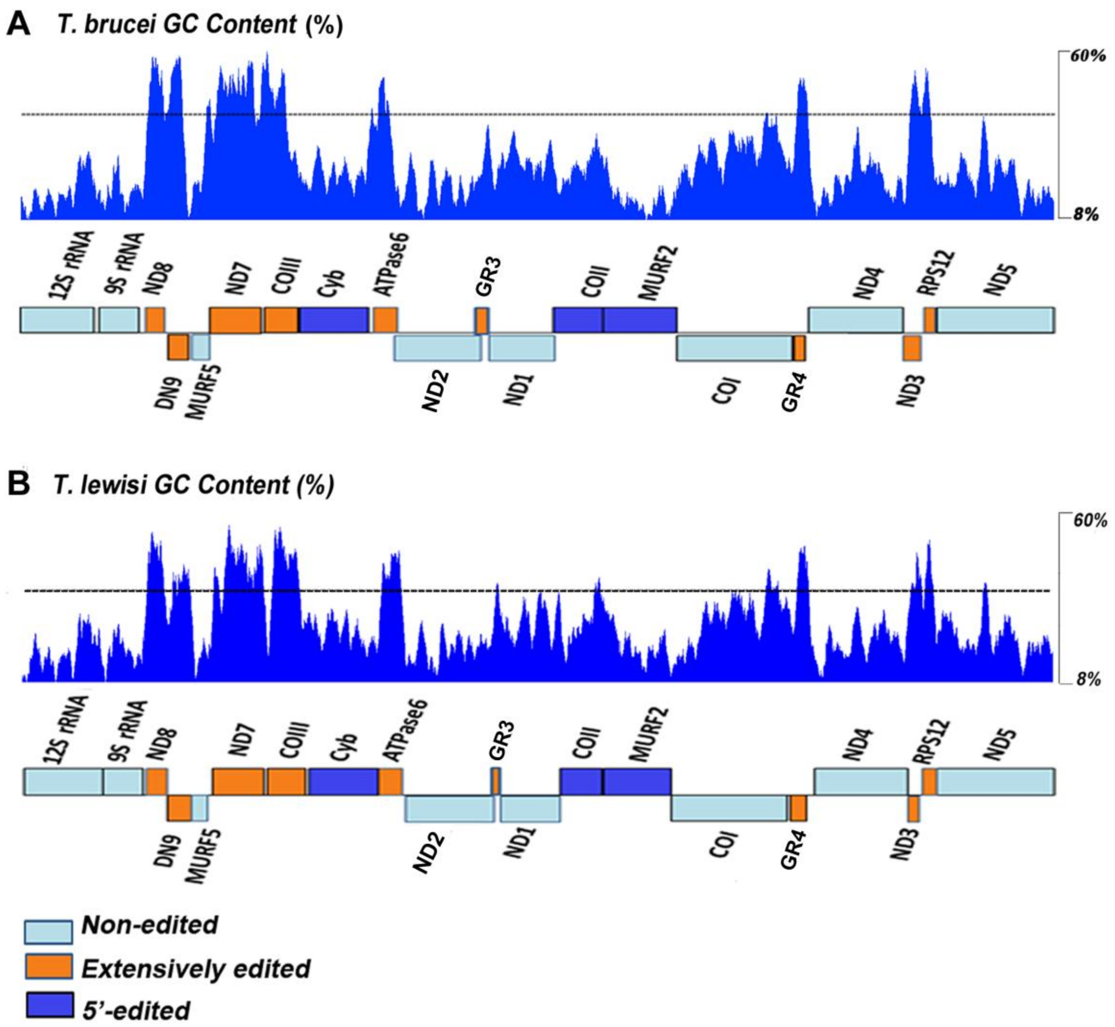

Additional file 5: Figure S5.

Graphs show the GC percentage of *T. brucei* (A) and *T. lewisi* (B) maxicircle coding regions. The regions where the percentage GC content value lies above the dashed lines are likely undergo RNA editing. The window size used for this analysis was100 nt.
